# Evaluating glaucoma surgeries in the MIGS context

**Published:** 2020

**Authors:** Deepika Dhingra, Shibal Bhartiya

**Affiliations:** * CShah Satnam Ji Speciality Hospitals, Sirsa, Haryana, India; ** Glaucoma Services, Fortis Memorial Research Institute, Gurugram, Haryana, India

**Keywords:** glaucoma, minimally invasive glaucoma surgery, MIGS, trabeculectomy, glaucoma drainage device

## Abstract

The challenges of glaucoma management are many: the disease is chronic, progressive, often asymptomatic, and very often, the quality of life and costs of treatment is unacceptable to the patient. This is true for both medical therapy and conventional glaucoma surgery. The choice of therapy, especially the transition from the former to the latter, is now being bridged by Minimally Invasive Glaucoma Surgeries (MIGS). Choosing from the several options now available in the surgical armamentarium requires a deeper understanding of the available modalities. This review aims to provide an overview of the decision-making process, keeping in mind age, type of glaucoma, life expectancy, socioeconomic status, patient expectations, and coexisting cataract.

## Introduction

Glaucoma surgeries, unlike cataract surgeries, are not one-time procedures. There is a frequent need of stringent postoperative follow-up for the management of various early and late post-operative complications and repeated procedures because of gradual decrease in the efficacy of procedure over time. Despite refinements of the surgical technique, and the introduction of newer surgical procedures, the outcomes of glaucoma surgery remain suboptimal. 

In the quest for the perfect glaucoma surgery, a lot of innovations and devices have come into market. These are collectively labelled MIGS (Minimally Invasive Glaucoma Surgery) and are ab-interno glaucoma procedures often sparing the conjunctiva, and thought to be less aggressive than the standard glaucoma surgical procedures, namely, trabeculectomy and glaucoma drainage devices (GDD) (**Table 1**) [**1**]. 

Even though surgical success criteria have been defined by WGA (World Glaucoma Association) consensus in 2011, the criteria for success of many surgeries have been variably defined in different trials (**Table 2**). Even over the last decade, there has also been a growing understanding that the criteria for success cannot be generalized and have too many variables. These include the purpose of procedure, its efficacy as well as safety, patient age, stage of glaucoma, etc. In a present scenario, there are many surgical options for patients as well as for surgeons and there is a demand of tailored management for each patient. This review aimed to critically evaluate each of these, making the surgical decision process a little less ambiguous. 

**Table 1:** Available MIGS devices and mechanism of action

Mechanism	Example	Outflow Pathway	FDA approval
Increase in trabecular outflow	1. iStent/ iStent inject	Implant is inserted through trabecular meshwork to Schlemm’s canal	• Mild-moderate open angle glaucoma (OAG) in conjunction with cataract surgery
	2. Hydrus implant		
	3. Ab-interno trabeculotomy with Trabectome device or Kahook dual blade	Removes the trabecular meshwork and inner wall of Schlemm’s canal	• Medically uncontrolled primary or secondary OAG with or without cataract extraction
	4. Gonioscopy assisted transluminal trabeculotomy (GATT)	Gonioscopic guided ab-interno trabeculotomy using microcatheter (iTrack) or sutures (prolene/ nylon) after performing a 1-2 mm goniotomy	
	5. Ab-interno canaloplasty using iTrack	Ab-interno viscodilatation of Schlemm’s canal	• Mild-moderate POAG with or without cataract surgery
	Cypass		• Cypass withdrawn from market
Increase in uveoscleral outflow	Solx Gold shunt	Implants are inserted into suprachoroidal space after creating a localized cyclodialysis	• Not yet approved
	iStent supra		• CE approved for mild-moderate POAG with or without cataract surgery, but not yet FDA approved
Subconjunctival filtration	Xen implant	Implant is inserted through trabecular meshwork to subconjunctival space	• Medically uncontrolled POAG, pseudoexfoliation or pigmentary glaucoma patients or refractory glaucoma after failed previous surgery with or without cataract surgery
	Innfocus		• Not yet approved
Decrease in aqueous humour production	Endolaser Cyclophotocoagulation	Ab-interno cyclophotocoagulation to ablate ciliary processes by direct visualization	• Refractory glaucoma with or without cataract surgery
			• In medically controlled glaucoma in combination with cataract surgery
			

**Table 2:** Criteria used for defining surgical success in evaluating glaucoma surgery

Surgery	Success criteria
Trabeculectomy	• IOP 6-21 mmHg and at least 30% IOP reduction [**2**]
	• IOP 5-21 mmHg and IOP decrease of ≥ 20% [**3**,**4**]
	• IOP ≥ 6 - ≤ 18 mmHg [**5**]
	• IOP > 5 - ≤ 18 mmHg or 20% reduction [**6**,**7**]
	• IOP 6-16 mmHg without medication (complete success), ≥ 6 - ≤ 16 mmHg with one antiglaucoma medicine or needling (Qualified success) [**8**]
	• IOP ≤ 15 mmHg [**9**]
Glaucoma Drainage Devices	• IOP ≤ 21 or > 20% IOP reduction, success rates at IOP levels ≤ 17, ≤ 14 mmHg also evaluated [**10**,**11**]
	• IOP ≤ 21, ≤ 16 mmHg [**12**], qualified success - up to 2 topicals
	• 5-21 or ≥ 25% IOP reduction [**13**]
	• IOP ≥ 5 - ≤ 18 mmHg [**14**]
Deep Sclerectomy	• IOP reduction > 25% [**15**]
	• IOP of < 18 mmHg and at least 20% IOP reduction [**16**]
MIGS	• IOP reduction ≥ 20% on the same or fewer medications in a study on Xen implant without cataract surgery [**17**]
	• IOP reduction and medicine reduction in a study on Trabeular bypass stents in combination with cataract surgery [**18**]

**Which surgery to choose?**

Glaucoma surgery is no longer considered the last-ditch effort to decrease intraocular pressure (IOP) in the end stage glaucoma, or in patients not effectively controlled by maximal medical treatment. Patients, and doctors alike, are increasingly considering surgery for patient comfort, when patients do not want to use eye drops, and choose a safer surgical option for a better quality of life. But not a single surgery fits every patient. The choice of surgery can be guided by several factors like:

**a. Desired IOP level:** Without doubt the single most important criteria to evaluate a glaucoma surgery remains its efficacy in controlling the IOP. That said, there is a need to realize that the criteria for success of IOP control ≤ 21 mmHg with or without medications (qualified or complete success respectively) might not be valid in each case. IOP level < 21 mmHg might not be good for moderate-advanced glaucoma or with Trab/ tube surgeries, whereas the same might be considered excellent for MIGS when performed in early glaucoma. Similarly, in cases of MIGS, if a patient still has to use medicines, when the surgery was performed early on in the disease to ensure freedom from eye drops, the procedure would be considered a failure.

**b. Type of glaucoma and age of the patient:**

**Early and Moderate Primary Open Angle Glaucoma (POAG)**

In early POAG, medical or laser treatment are usually effective options, however, some patients who are intolerant/ non-compliant to medicines or not very well controlled with medical/ laser treatment, MIGS (for example iStent, Trabectome, Hydrus, endolaser cyclophotocoagulation) are good surgical options in terms of reduction in IOP, as well as the number of glaucoma medications, especially since they are conjunctiva sparing and have lesser complications. However, some MIGS devices are ab-interno procedures but are implanted subconjunctivally like Xen implant or Innfocus, which might not be the first choice in these patients, as they involve conjunctiva and have bleb related complications similar to trabeculectomy. 

Trabeculectomy may also not be a good option in these cases because of the significantly higher risk of post-operative complications, increase in cataract formation, higher failure rates because of strong healing response in young [**19**]. If the trabeculectomy in this scenario results in qualified success with persistent need of medicines, it would actually be considered a failure.

In a young patient, with early glaucoma, safety of a procedure is the main priority and the procedure has to be effective on long-term without vision threatening complications. Conjunctiva sparing surgeries should be preferred so as to preserve the conjunctiva for future surgery if needed. At present, there is no ideal surgery that is safe and conjunctival sparing with long-term efficacy. This is especially relevant since the long-term outcome with MIGS is yet to be ascertained [**20**,**21**].

On the other hand, in elderly patients with limited life expectancy, MIGS can be offered with good safety profile over trabeculectomy. Non-penetrating deep sclerectomy (NPDS) is another safer option with similar success rates as of trabeculectomy, but with fewer complications [**22**,**23**].

**Severe POAG**

Severe cases usually require lower target IOPs and MIGS might not be a good option in these cases as many of the stents (Schlemm canal based) are unable to maintain lower levels of IOP because of downstream resistance and episcleral venous pressure [**24**]. Trabeculectomy and deep sclerectomy, or even suprachoroidal shunts, may be better surgical options in these cases. 

Primary tube surgery is considered another good option in view of good success rates with fewer complications compared to trabeculectomy (Trab) with Mitomycin-C (MMC), as has been reported in Primary Tube versus Trabeculectomy (TVT) study (overall success 81% vs. 92% and complete success 14% vs. 59% respectively, in Baerveldt implant versus Trab MMC, low rate of serious vision threatening complications or reoperation for complications like hypotony maculopathy, bleb leak, hyphaema (1% vs. 7% respectively) [**11**].

**Secondary glaucomas**

They are a heterogenous group and the surgical results in each of these may vary as per etiology. In the absence of active inflammation, and in patients with healthy conjunctiva, Trab MMC has good success rates as in steroid induced, traumatic and post keratoplasty glaucoma (73% success rate at 22 months) [**25**], however, in neovascular glaucoma (NVG) or uveitic glaucoma, trabeculectomy has poor success rates (overall success rate 54%, 9% complete, 45% qualified in NVG) [**26**] and GDDs are more useful in these cases. MIGS are not good options in these cases although steroid induced glaucoma or pseudoexfoliative glaucoma may be managed with MIGS; currently, there is insufficient literature on their efficacy. 

**Angle closure disease**

Angle closure glaucoma is not amenable to treatment by MIGS unless the angle is adequately open after iridotomy/ iridoplasty/ cataract extraction. These patients can be managed with cataract extraction or trabeculectomy in isolation or combined phacotrabeculectomy. 

**Co-existent cataract and glaucoma**

In angle closure disease, cataract surgery alone helps in IOP control, however, in few cases of angle closure disease and in open angle glaucoma, combined surgery is required [**27**]. In combination with cataract surgery, MIGS have been reported to have good success rates and can be preferred in both types of glaucoma if the angle is fairly open to visualize structures, whereas phacotrabeculectomy is less often performed and less preferred because of longer surgical time, high chances of intraoperative complications and poor success rates compared to stepwise surgery [**28**].

**c. Surgeon expertise:** Contrary to popular opinion, MIGS procedures have a relatively steep learning curve. They require training in direct gonioscopy, clear identification of angle structures and hands on training for angle-based procedures. Similarly, the deep sclerectomy procedure, which is often considered safer compared to conventional trabeculectomy because of its non-penetrating nature, also has a steep learning curve. The procedure may be complicated by inadvertent rupture of Descemet’s membrane (3.5-7% of the cases) changing the procedure to a penetrating one, similar to Trab [**29**].

**d. Feasibility of procedure and contraindications:** There are several conditions when MIGS cannot be used, as in cases with poor visibility of the angle because of extensive anterior synechiae, corneal opacity, and ocular surface disease. Surgery is difficult in patients with narrow palpebral apertures, cervical spine abnormalities, and in unco-operative patients who are not able to follow commands. 

Many of these procedures including iStent and Hydrus are approved in conjunction with cataract surgery, which may not be appropriate in young patients or patients having very early cataract. So, in these cases, conventional trabeculectomy is still the first choice of surgery, however, it must be kept in mind that trabeculectomy surgery leads to progression of cataract [**30**]. There are several MIGS, including Trabectome, Xen implant, gonioscopy assisted transluminal trabeculotomy, which can be performed alone without cataract surgery. Thus, an informed decision has to be made in these circumstances after a discussion with the patient. Availability of devices and the additional cost of the surgery represent another major concern with regard to the feasibility of MIGS.

**e. Safety of the procedure and complications:** Safety is of utmost importance and risk-benefit ratio must be considered in deciding the type of procedure. The complications that are relatively more acceptable in patients with advanced disease, might not be so in patients with an early disease. Therefore, complications that may be considered acceptable can vary according to the procedures. MIGS and deep sclerectomy are considered safer options and MIGS are better in terms of sparing the conjunctiva and avoiding the use of anti-metabolites. Similarly, hypotony after DS may be acceptable as it doesn’t lead to hypotony maculopathy, however, this may not be the case with trabeculectomy. Though MIGS procedures are presumed to have lesser complications, they can have unique complications of stent malpositioning or obstruction apart from the complications of immediate postoperative rise in IOP, hyphaema, hypotony, etc., which are usually transient and not vision threatening. If the same procedure results in a complication that can have a long-term impact on vision, it should not be acceptable. Because of this reason, Cypass shunt was voluntarily withdrawn from the market by Alcon in 2018, because of unacceptably high endothelial cell loss even though a significant loss occurs with trabeculectomy and GDD procedures as well [**31**]. **Table 3** summarizes the complications associated with different procedures.

**Table 3:** Complications following glaucoma surgery: A comparative overview

	Trab	Trab with express shunt	Baerveldt	AGV	NPDS	MIGS
Hypotony	16.8 - 39.3% [**32**-**34**]	10.5% [**35**]	13% [**32**,**36**]	2% [**36**]	4.3-9.9% [**34**,**37**]	13.8% with Cypass [**38**] 15.3% with Xen [**39**]
Hypotony maculopathy	5.18% [**35**]	3.17% [**35**]	1% [**32**]	Rare reports [**40**,**41**]	0-2.1% [**42**,**43**]	1.08% with Xen [**44**] 1.3% with Cypass [**45**]
Hyphaema	14.9-17.2% [**33**,**34**]	1.6% [**35**]	5% [**46**]	18.3% [**33**]	7.4-12.4% [**34**,**37**]	24.3% with Xen [**47**] 0.02% for iStent [**48**,**49**] 19.04% for Hydrus [**50**] 2.7% for Cypass [**45**]
Shallow anterior chamber	11.8-32.1% [**33**,**34**]	4.74% [**35**]	3% [**46**]	11.11% [**33**]	2.9-8.9% [**34**,**37**]	0-2.3% [**51**]
Choroidal detachment	3.2-15.9% [**34**,**52**]	10.38% [**35**]	3% [**46**]	12% [**53**]	8.6-10.2% [**34**,**37**]	15.3% with Xen [**39**]
Progressive cataract	29-35% [**30**,**34**]	12% at 2 years [**54**]	8% [**36**]	8% [**36**]	6.6-12.7% [**34**,**37**]	12.2% with Cypass [**55**] 11.1% with iStent [**56**]
Loss of light perception	2% [**32**]	3.2% [**57**]	26% [**36**]	12% [**36**]		No reports yet [**17**]
Bleb leak	6.7-13.6% [**33**,**35**,**44**]	16.8% [**35**]				1.93% with Xen [**44**]
Endophthalmitis	1.6% [**35**]	1.6% [**35**]	0.5-1.4% [**58**,**59**]	1.7% [**60**]	Rare, isolated reports of blebitis [**61**]	0.5% with Xen [**62**]
Endothelial cell loss	-3±8% to 9.6% at 1 year [**39**, **63**]	-10±8% at 12 months [**39**]	Mainly with anterior chamber implantation 7.2% at 6 months 12% at 1 year [**64**] 4.54% per year [**65**]	9% at 6 months to 12% at 1 year [**66**]	4.5% at 1 year [**63**]	2.1% in one month with Xen implant in cases with dynamic corneal contact [**67**], 18.4% with Cypass at 5 years in Compass-XT study (unpublished data)
Stent malpositioning						12.2% with Xen [**47**]
Stent obstruction						4% with iStent [**68**] 2.4-5.4% with Cypass [**38**,**45**]
Need of needling	23-30.76% over 3 years [**44**,**69**]	15.9% over 30 months follow-up [**69**]				43.24% [**44**]
Need of Re-surgery	7-28% over a 5-year period [**53**,**70**]	5.12% during 1st year [**71**], 30.6% at 3 years [**72**]	5.4–17% over 5 years [**33**,**73**]	17–40% over 5-year period [**36**,**53**]	3.7–5.4% after 1–3 years [**74**,**75**]	14.1% with Xen after 12 months of surgery [**17**] 4.3% with InnFocus at 3 years [**76**] 7.4% with iStent [**39**]

**f. Survival of surgery:** The longevity and survival of the procedure is yet another confounding factor. For surgeries that are minimally invasive and repeatable, the longevity thought extremely desirable, is not absolutely imperative. Especially since a lot of these surgeries are conjunctiva sparing, they do not preclude more definitive surgeries at a later date. For example, iStent has been reported to decrease IOP of up to 40% at 1 year and 16.3% at 5 years with reduction in medication load in up to 85% of the cases at 1 year and 43% at 5 years [**77**,**78**]. Thus, if they offer a few drug free years to the patient, they must be considered successes. Survival of surgery on long-term is of less importance when choosing it for elderly patients with a limited life expectancy, where their quality of life impact and morbidity should be considered more important.

**g. Ancillary procedures:** Procedures like needling and goniopuncture are not considered failures of trabeculectomy and NPDS respectively, but merely ancillary procedures. Similarly, the hypertensive phase of the Ahmed Glaucoma valve (AGV) is considered par for course, as is the two-stage surgery for Baerveldt like devices. In the case of MIGS, a second surgery may also be similarly considered an ancillary procedure since the surgery is not very invasive, except for the significant costs involved. In case of MIGS, multiple MIGS with different sites of action can be performed simultaneously or sequentially as a safer alternative to a more invasive glaucoma surgery [**79**].

**h. Health economics:** Cost effectiveness is yet other criteria that must be kept in mind when judging a procedure. For a glaucoma surgery, the cost factor has to be considered in terms of cost of the surgery, efficacy of the procedure in terms of the decrease in drugs use or follow-up visits and the comparison of the cost of one-time procedure with the overall cost of drugs for that period of time. For example, adding the express shunt to trabeculectomy increases its cost significantly, does not increase its longevity, and in fact, turns a surgery that can be used in both POAG and PACG into a surgery that can only be used in open angles [**7**]. Thus, its success criteria need to be significantly more stringent. Another thing to remember concerning the health economics of glaucoma is the high cost of the minimally invasive devices. Unless they result in a significant economic saving by significantly decreasing or eliminating the use of anti-glaucoma medication for a significant period of time, or decreasing postoperative follow-ups, their use cannot be justified in terms of costs. Trabecular bypass shunts have been shown to be cost effective over standard care with improvement in quality of life [**80**]. Long-term data regarding cost-effectiveness is currently inexistent. Trabeculectomy is still the cheapest surgical option amongst all glaucoma procedures and the most commonly performed procedure [**81**,**82**].

**i. Quality of Life, QoL:** The QoL impact of each of these surgeries must also be part of the success criteria. Bleb dysesthesias, multiple interventions by needlings and complicated treatment regimens postoperatively, complication rates, etc., may significantly affect the QoL of a patient following trabeculectomy. Therefore, its use in a patient with early disease may not be justified. Regarding MIGS, QoL data is not available as there are limited studies at present. A study has reported comparable Qol in patients undergoing MIGS (iStent, Trabectome) or trabeculectomy at 6 months postoperatively, however, better social functioning, color vision, and postoperative day 1 visual acuity, were seen in MIGS group, but the trabeculectomy group required lesser topical antiglaucoma medicines [**83**].

**Consensus amongst glaucoma surgeons**

A survey of American Glaucoma society (AGS) published in 2017 for preferred practice preferences for the type of glaucoma surgery among AGS members has also reported that Trab MMC, GDD and MIGS constituted 59±30%, 23±23% and 14±20% respectively as an initial surgery in POAG. Although the use of GDD surgery has increased and trabeculectomy has decreased from 1996 to 2016 [**81**], Trab MMC was still the most popular primary glaucoma surgery among surgeons. 

MIGS are emerging devices that are expected to have improved safety, however, there is a need of more randomized controlled trials and long-term results are awaited. They definitely have a future in patients who are non-compliant or intolerant to medications and can act as a bridge between medical/ laser treatment and conventional glaucoma surgeries, having different indications compared to conventional glaucoma surgeries. A tailored approach depending on the patient profile, type of glaucoma, and life expectancy can help in the better management of glaucoma patients.

## Conclusion 

Glaucoma is a continuum from early to end stage, thus each surgery has a unique space with respect to success and failure criteria applicable. With the introduction of the newer surgical procedures, the algorithm for the choice glaucoma surgery has become more complex. However, this also implies that better and safer surgical procedures, which may be better suited to the needs of the individual, are now available. In addition, a combination of surgical procedures may be performed for any patient during his or her clinical lifetime in order to best preserve their quality of life.

**Sources of funding**

None.

**Disclosures**

Authors have nothing to disclose.

**Conflict of interest**

None of the authors have any conflict of interest.

## References

[1] Ansari E (2017). An update on implants for minimally invasive glaucoma surgery (MIGS). Ophthalmol Ther.

[2] Vahedian Z, Mafi M, Fakhraie G, Zarei R, Eslami Y, Ghadimi H, Mohebbi M (2017). Short-term Results of Trabeculectomy Using Adjunctive Intracameral Bevacizumab Versus Mitomycin C: A Randomized Controlled Trial. J Glaucoma.

[3] El-Sayyad F, El-Saied HMA, Abdelhakim MASE (2017). Trabeculectomy with Ologen versus Mitomycin C in Juvenile Open-Angle Glaucoma: A 1-Year Study. Ophthalmic Res.

[4] Tanna AP, Rademaker AW, de Moraes CG, Godfrey DG, Sarkisian SR Jr, Vold SD, Ritch R (2016). Collagen matrix vs mitomycin-C in trabeculectomy and combined phacoemulsification and trabeculectomy: a randomized controlled trial. BMC Ophthalmol.

[5] Wlaź A, Wilkos-Kuc A, Rozegnał-Madej A, Żarnowski T (2019). Phacotrabeculectomy using collagen matrix implant (Ologen(®) ) versus mitomycin C: a prospective randomized controlled trial. Acta Ophthalmol.

[6] Kaushik J, Parihar JK, Jain VK, Gupta S, Nath P, Durgapal P, Ram J (2017). Efficacy of Bevacizumab Compared to Mitomycin C Modulated Trabeculectomy in Primary Open Angle Glaucoma: A One-Year Prospective Randomized Controlled Study. Curr Eye Res.

[7] Gonzalez-Rodriguez JM, Trope GE, Drori-Wagschal L, Jinapriya D, Buys YM (2016). Comparison of trabeculectomy versus Ex-PRESS: 3-year follow-up. Br J Ophthalmol.

[8] Yadava U, Jaisingh K, Dangda S, Thacker P, Singh K, Goel Y (2017). Simultaneous use of amniotic membrane and Mitomycin C in trabeculectomy for primary glaucoma. Indian J Ophthalmol.

[9] Sihota R, Angmo D, Chandra A, Gupta V, Sharma A, Pandey RM (2015). Evaluating the long-term efficacy of short-duration 0.1 mg/ml and 0.2 mg/ml MMC in primary trabeculectomy for primary adult glaucoma. Graefes Arch Clin Exp Ophthalmol.

[10] Islamaj E, Wubbels RJ, de Waard PWT (2018). Primary Baerveldt versus trabeculectomy study after one-year follow-up. Acta Ophthalmol.

[11] Gedde SJ, Feuer WJ, Shi W, Lim KS, Barton K, Goyal S, Ahmed IIK, Brandt J (2018). Primary Tube Versus Trabeculectomy Study Group. Treatment Outcomes in the Primary Tube Versus Trabeculectomy Study after 1 Year of Follow-up. Ophthalmology.

[12] Nilforushan N, Yadgari M, Jazayeri AA, Karimi N (2016). Evaluation of success after second Ahmed glaucoma valve implantation. Indian J Ophthalmol.

[13] Marchini G, Ceruti P, Vizzari G, Toscani M, Amantea C, Tosi R, Marchetti P (2016). Long-term Outcomes of a Modified Technique Using the Baerveldt Glaucoma Implant for the Treatment of Refractory Glaucoma. J Glaucoma.

[14] Pandav SS, Seth NG, Thattaruthody F, Kaur M, Akella M, Vats A (2019). Long-term outcome of low-cost glaucoma drainage device (Aurolab aqueous drainage implant) compared with Ahmed glaucoma valve. Br J Ophthalmol.

[15] Harju M, Suominen S, Allinen P, Vesti E (2018). Long-term results of deep sclerectomy in normal-tension glaucoma. Acta Ophthalmol.

[16] Mansouri K, Tran HV, Ravinet E, Mermoud A (2010). Comparing deep sclerectomy with collagen implant to the new method of very deep sclerectomy with collagen implant: a single-masked randomized controlled trial. J Glaucoma.

[17] Grover DS, Flynn WJ, Bashford KP, Lewis RA, Duh YJ, Nangia RS, Niksch B (2017). Performance and Safety of a New Ab Interno Gelatin Stent in Refractory Glaucoma at 12 Months. Am J Ophthalmol.

[18] Best UP, Domack H, Schmidt V, Khalifa M (2019). Microinvasive glaucoma surgery-Efficacy of trabecular stents in combined interventions: A clinical study on 65 eyes. Ophthalmology.

[19] Gressel MG, Heuer DK, Parrish RK 2nd (1984). Trabeculectomy in young patients. Ophthalmology.

[20] Le JT, Bicket AK, Wang L, Li T (2019). Ab interno trabecular bypass surgery with iStent for open-angle glaucoma. Cochrane Database Syst Rev.

[21] King AJ, Shah A, Nikita E, Hu K, Mulvaney  CA, Stead R, Azuara-Blanco  A (2018). Subconjunctival draining minimally-invasive glaucoma devices for medically uncontrolled glaucoma. Cochrane Database Syst Rev.

[22] El Sayyad F, Helal M, El-Kholify H, Khalil M, El-Maghraby A (2000). Nonpenetrating deep sclerectomy versus trabeculectomy in bilateral primary open-angle glaucoma. Ophthalmology.

[23] Ambresin A, Shaarawy T, Mermoud A (2002 ). Deep sclerectomy with collagen implant in one eye compared with trabeculectomy in the other eye of the same patient. J Glaucoma.

[24] Al-Mugheiry TS, Cate H, Clark A, Broadway DC (2017). Microinvasive glaucoma stent (MIGS) surgery with concomitant phacoemulsification cataract extraction: Outcomes and the learning curve. J Glaucoma.

[25] Ishioka M, Schimezaki J, Jamagami J, Fujishima H, Shimmura S, Tsubota K (2000). Trabeculectomy with mitomycin C for postkeratoplasty glaucoma. Br. J Ophthalmol.

[26] Sisto D, Vetrugno M, Trabucco T, Cantatore F, Ruggeri G, Sborgia C (2007). The role of antimetabolites in filtration surgery for neovascular glaucoma: Intermediate-term follow-up. Acta Ophthalmol Scand.

[27] Pandav SS, Seth NG, Arora A, Thattaruthody F, Jurangal A, Kaushik S, Raj S (2019 ). Intraocular pressure reduction in a spectrum of angle closure disease following cataract extraction. Indian J Ophthalmol.

[28] El Sayed YM, Elhusseiny AM, Albalkini AS, El Sheikh RH, Osman MA (2019). Mitomycin C-augmented Phacotrabeculectomy Versus Phacoemulsification in Primary Angle-closure Glaucoma: A Randomized Controlled Study. J Glaucoma.

[29] Karaconji T, Mercieca K, Romera P, McNaught A, Anand N (2019). A comparison of deep sclerectomy trainer versus trainee outcomes. J Glaucoma.

[30] Casson R, Rahman R, Salmon JF (2001). Long term results and complications of trabeculectomy with low dose mitomycin C in patients at risk of filtration failure. Br J Ophthalmol.

[31] Moschos MM, Panos GD, Lavaris A, Droutsas K, Gatzioufas Z (2017 ). Trabeculectomy with or without Anterior Chamber Maintainer: A Study on Intraocular Pressure, Endothelial Cells, and Central Corneal Thickness. Semin Ophthalmol.

[32] Gedde SJ, Schiffman JC, Feuer WJ, Herndon LW, Brandt JD, Budenz DL (2012 ). Tube Versus Trabeculectomy Study Group. Treatment outcomes in the Tube Versus Trabeculectomy (TVT) Study after five years of follow-up. Am J Ophthalmol.

[33] Haibo T, Xin K, ShiHeng L, Lin L (2015). Comparison of Ahmed glaucoma valve implantation and trabeculectomy for glaucoma: A systematic review and meta-analysis. PLoS One.

[34] Cheng JW, Xi GL, Wei RL, Cai JP, Li Y (2009). Efficacy and tolerability of nonpenetrating glaucoma surgery augmented with mitomycin C in treatment of open-angle glaucoma: a meta-analysis. Can J Ophthalmol.

[35] Wang W, Zhou M, Huang W, Zhang X (2013). Ex-PRESS implantation versus trabeculectomy in uncontrolled glaucoma: a meta-analysis. PLoS One.

[36] Budenz DL, Barton K, Gedde SJ, Feuer WJ, Schiffman J, Costa VP (2015). Five-year treatment outcomes in the Ahmed Baerveldt comparison study. Ophthalmology.

[37] Cheng JW, Cheng SW, Cai JP, Li Y, Wei RL (2011). Systematic review of the efficacy of nonpenetrating glaucoma surgery in the treatment of open angle glaucoma. Med Sci Monit.

[38] Hoeh H, Vold SD, Ahmed IK, Anton A, Rau M, Singh K, Chang DF, Shingleton BJ, Lanchulev T (2016). Initial clinical experience with the CyPass micro-stent: Safety and surgical outcomes of a novel supraciliary microstent. J Glaucoma.

[39] Galal A, Bilgic A, Eltanamly R, Osman A (2017). Xen glaucoma implant with mitomycin-C 1-year follow-up: result and complications. J Ophthalmol.

[40] Canut MI, Alonso-Agesta M, Botella J, Julio G (2019). Cauterized suture for complete tube occlusion of Ahmed glaucoma valve in hypotony maculopathy. Eur J Ophthalmol.

[41] Lin M, Alizadeh R, Law SK (2019). Outcomes of Combined Ahmed Glaucoma Valve and Trabeculectomy Revision with Adjunctive Antimetabolite. J Glaucoma.

[42] Suominen S, Harju M, Ihanamäki T, Vesti E (2010). The effect of deep sclerectomy on intraocular pressure of normal-tension glaucoma patients: 1-year results. Acta Ophthalmol.

[43] Al Obeidan SA (2015). Incidence, efficacy and safety of YAG laser goniopuncture following nonpenetrating deep sclerectomy at a university hospital in Riyadh, Saudi Arabia. Saudi J Ophthalmol.

[44] Schlenker MB, Gulamhusein H, Conrad-Hengerer I, Somers A, Lenzhofer M, Stalmans I, Reitsamer H, Hengerer FH, Ahmed IIK (2017 ). Efficacy, Safety, and Risk Factors for Failure of Standalone Ab Interno Gelatin Microstent Implantation versus Standalone Trabeculectomy. Ophthalmology.

[45] Vold S, Ahmed IK, Craven ER, Mattox C, Stamper R, Packer M, Brown RH, Ianchulev T (2016). CyPass Study Group. Two-year COMPASS trial results: supraciliary microstenting with phacoemulsification in patients with open angle glaucoma and cataracts. Ophthalmology.

[46] Namavari A, Hyde RA, Wang D, Vajaranant TS (2016). Primary Baerveldt shunt implantation: Outcomes and complications. Ophthalmol Ther.

[47] De Gregorio A, Pedrotti E, Russo L, Morselli S (2018). Minimally invasive combined glaucoma and cataract surgery: clinic results of the smallest ab interno gel stent. Minimally invasive combined glaucoma and cataract surgery: clinic results of the smallest ab interno gel stent.

[48] Patel I, de Klerk TA, Au L (2013). Manchester iStent study: early results from a prospective UK case series. Clin Experiment Ophthalmol.

[49] Belovay GW, Nagi A, Chan BJ, Rateb M, Ahmed II (2012). Using multiple trabecular micro-bypass stents in cataract patients to treat open-angle glaucoma. J Cataract Refract Surg.

[50] Gandolfi SA, Ungaro N, Ghirardini S, Tardini MG, Mora P (2016). Comparison of surgical outcomes between canaloplasty and Schlemm’s canal scaffold at 24 months’ follow-up. J Ophthalmol.

[51] Brandão LM, Grieshaber MC (2013). Update on Minimally Invasive Glaucoma Surgery (MIGS) and New Implants. J Ophthalmol.

[52] Zhou M, Wang W, Huang W, Zhan X (2014). Trabeculectomy with versus without releasable sutures for glaucoma-A meta-analysis of randomized controlled trials. BMC Ophthalmol.

[53] Tran DH, Souza C, Ang MJ, Loman J, Law SK, Coleman AL, Caprioli J (2009 ). Comparison of long-term surgical success of Ahmed valve implant versus trabeculectomy in open-angle glaucoma. Br J Ophthalmol.

[54] Arimura S, Miyake S, Iwasaki K, Gozawa M, Matsumura T, Takamura Y, Inatani M (2018). Randomised Clinical Trial for Postoperative Complications after Ex-PRESS Implantation versus Trabeculectomy with 2-Year Follow-Up. Sci Rep.

[55] Garcia—Feijoo J, Rau M, Grisanti S, Grisanti S, Hoh H, Erb C, Guguchkova P, Ahmed I, Grabner G, Reitsamer H (2015). Supraciliary micro-stent implantation for open-angle glaucoma failing topical therapy: 1-year results of a multicenter study. Am J Ophthalmol.

[56] Popovic M, Campos-Moller X, Saheb H, Ahmed IIK (2018 ). Efficacy and Adverse Event Profile of the iStent and iStent Inject Trabecular Micro-bypass for Open-angle Glaucoma: A Meta-analysis. J Curr Glaucoma Pract.

[57] Waisbourd M, Fischer N, Shalev H, Spierer O, Artsi EB, Rachmiel R, Shemesh G, Kurtz S (2016). Trabeculectomy with Ex-PRESS implant versus Ahmed glaucoma valve implantation-a comparative study. Int J Ophthalmol.

[58] Harbick KH, Sidoti PA, Budenz DL, Venkatraman A, Bruther M, Grayson DK, Ko A, Yi GN (2006). Outcomes of inferonasal Baerveldt glaucoma drainage implant surgery. J Glaucoma.

[59] Tsai JC, Johnson CC, Kammer JA, Dietrich MS (2006). The Ahmed shunt versus the Baerveldt shunt for refractory glaucoma II: longer-term outcomes from a single surgeon. Ophthalmology.

[60] Al-Torbak AA, Al-Shahwan S, Al-Jadaan I, Al-Hommadi A, Edward DP (2005). Endophthalmitis associated with the Ahmed glaucoma valve implant. Br J Ophthalmol.

[61] Wallin OJ, Montan PG (2007). Blebitis after deep sclerectomy. Eye.

[62] Karimi A, Lindfield D, Turnbull A, Dimitriou C, Bhatia B, Radwan M, Gouws P, Hanifudin A, Amerasinghe N, Jacob A (2019). A multi-centre interventional case series of 259 ab-interno Xen gel implants for glaucoma, with and without combined cataract surgery. Eye (Lond).

[63] Arnavielle S, Lafontaine PO, Bidot S, Creuzot-Garcher C, D'Athis P, Bron AM (2007). Corneal endothelial cell changes after trabeculectomy and deep sclerectomy. J Glaucoma.

[64] Iwasaki K, Arimura S, Takihara Y, Takamura Y, Inatani M (2018). Prospective cohort study of corneal endothelial cell loss after Baerveldt glaucoma implantation. PLoS One.

[65] Tan AN, Webers CA, Berendschot TT, de Brabander J, de Witte PM, Nuijts RM, Schouten JS, Beckers HJ (2017). Corneal endothelial cell loss after Baerveldt glaucoma drainage device implantation in the anterior chamber. Acta Ophthalmol.

[66] Kim MS, Kim KN, Kim CS (2016). Changes in Corneal Endothelial Cell after Ahmed Glaucoma Valve Implantation and Trabeculectomy: 1-Year Follow-up. Korean J Ophthalmol.

[67] Gillmann K, Bravetti GE, Mermoud A, Mansouri K (2019). Anterior Chamber XEN Gel Stent Movements: The Impact on Corneal Endothelial Cell Density. J Glaucoma.

[68] Samuelson TW, Katz LJ, Wells JM, Duh YJ, Giamporcaro JE (2011). US iStent Study Group. Randomized evaluation of the trabecular micro-bypass stent with phacoemulsification in patients with glaucoma and cataract. Ophthalmology.

[69] Tojo N, Otsuka M, Hayashi A (2018 ). Conventional trabeculectomy versus trabeculectomy with the Ex-PRESS(®) mini-glaucoma shunt: differences in postoperative interventions. Clin Ophthalmol.

[70] Landers J, Martin K, Sarkies N, Bourne R, Watson P (2011). A twenty-year follow-up study of trabeculectomy: risk factors and outcomes. Ophthalmology.

[71] Moisseiev E, Zunz E, Tzur R, Kurtz S, Shemesh G (2015). Standard Trabeculectomy and Ex-PRESS Miniature Glaucoma Shunt: A Comparative Study and Literature Review. J Glaucoma.

[72] Rabkin-Mainer Z, Wolf  A, Mathalone N, Melamud A, Buckman G, Edmunds B, Stein N, Steinberg DM, Geyer O (2018). Ex-PRESS Miniature Glaucoma Shunt Versus Ahmed Glaucoma Valve in the Surgical Treatment of Glaucoma in Pseudophakic Patients. J Glaucoma.

[73] Yalvac IS, Nurozler A, Kahraman C, Kasim R, Duman S (1998). The results of trabeculectomy with and without Mitomycin C in young patients. Ophthalmologica.

[74] Montolío Marzo S, Lanzagorta Aresti A, Davó Cabrera JM, Alfonso Muñóz EA, Piá Ludeña JV, Palacios Pozo E (2019). Malignant glaucoma after XEN45 implant. Arch Soc Esp Oftalmol.

[75] Heidinger A, Schwab C, Lindner E, Riedl R, Mossböck G (2019). A retrospective study of 199 xen45 stent implantations from 2014 to 2016. J Glaucoma.

[76] Batlle JF, Fantes F (2016). Three-Year Follow-up of a Novel Aqueous Humor MicroShunt. J Glaucoma.

[77] Voskanyan L, García-Feijoó J, Belda JI, Fea A, Jünemann A, Baudouin C (2014). Synergy Study Group. Prospective, unmasked evaluation of the iStent inject system for open- angle glaucoma: Synergy trial. Adv Ther.

[78] Arriola-Villalobos P, Martínez-de-la-Casa JM, Díaz-Valle D, Fernández-Pérez C, García-Sánchez J, García-Feijoó J (2012). Combined iStent trabecular micro-bypass stent implantation and phacoemulsification for coexistent open-angle glaucoma and cataract: a long-term study. Br J Ophthalmol.

[79] Bloom P, Au L (2018). Minimally invasive glaucoma surgery (MIGS) is a poor substitute for trabeculectomy- The great debate. Ophthalmol Ther.

[80] Patel V, Ahmed I, Podbielski D, Falvey H, Murray J, Goeree R (2019). Cost-effectiveness analysis of standalone trabecular micro-bypass stents in patients with mild-to-moderate open-angle glaucoma in Canada. J Med Econ.

[81] Vinod K, Gedde SJ, Feuer WJ, Panarelli JF, Chang TC, Chen PP, Parrish RK 2nd (2017 ). Practice preferences for glaucoma surgery: A survey of the American Glaucoma Society. J Glaucoma.

[82] Kaplan RI, De Moraes CG, Cioffi GA, Al-Aswad LA, Blumberg DM (2015). Comparative Cost-effectiveness of the Baerveldt Implant, Trabeculectomy With Mitomycin, and Medical Treatment. JAMA Ophthalmol.

[83] Pahlitzsch M, Klamann MK, Pahlitzsch ML, Gonnermann J, Torun N, Bertelmann E (2017). Is there a change in the quality of life comparing the micro-invasive glaucoma surgery (MIGS) and the filtration technique trabeculectomy in glaucoma patients?. Graefes Arch Clin Exp Ophthalmol.

